# Improving the Sensing Properties of Graphene MEMS Pressure Sensor by Low-Temperature Annealing in Atmosphere

**DOI:** 10.3390/s22208082

**Published:** 2022-10-21

**Authors:** Daosen Liu, Shengsheng Wei, Dejun Wang

**Affiliations:** 1 Liaoning Key Lab of Integrated Circuit and Biomedical Electronic System, Faculty of Electronic Information and Electrical Engineering, Dalian University of Technology, Dalian 116024, China; 2 Communication and Electronic Engineering Institute, Qiqihar University, Qiqihar 161006, China

**Keywords:** MEMS, graphene monolayer, pressure sensing, annealing

## Abstract

The high demand for pressure devices with miniaturization and a wide bearing range has encouraged researchers to explore new high-performance sensors from different approaches. In this study, a sensitive element based on graphene in-plane compression properties for realizing pressure sensing is experimentally prepared using microelectromechanical systems (MEMS) fabrication technology; it consists of a 50 µm thick, 1400 µm wide square multilayer component membrane and a graphene monolayer with a meander pattern. The prepared sample is extensively characterized and analyzed by using various techniques, including atomic force microscopy, Raman spectroscopy, infrared spectroscopy, X-ray photoelectron spectroscopy, COMSOL finite element method, and density functional theory. The sensing performance of the new pressure sensor based on the sensitive element are obtained by theoretical analysis for electromechanical measurements of the sensitive element before and after low-temperature annealing in atmosphere. Results demonstrate that atmospheric annealing at 300 °C enhances the pressure sensing sensitivity by 4 times compared to pristine graphene without annealing, which benefits from the desorption of hydroxyl groups on the graphene surface during annealing. The sensitivity is comparable and even better than that of previous sensors based on graphene in-plane properties. Our results provide new insights into realizing high-performance MEMS devices based on 2D sensitive materials.

## 1. Introduction

Graphene, experimentally discovered in 2004 [1,2], is the most robust and flexible material at present. Studies on graphene have attracted considerable scientific interest [3,4,5,6] and it has become an important topic. A number of excellent properties, such as ultrahigh electrical conductivity, carrier mobility, and thermal conductivity, have been found theoretically and experimentally [7,8,9,10], making graphene a promising material for improving the sensitivity, response speed, and thermal conductivity of electronic devices. A great amount of effort has been exerted toward realizing the application of graphene in electronic devices, such as in flexible touch screens [11], high-frequency transistors [12], and high-sensitivity sensors [13,14,15,16]. Studies on graphene microelectromechanical system (MEMS) pressure sensors have attracted a certain degree of attention [17,18,19,20,21,22], as this would be advantageous for the miniaturization and batch production of sensors. Such sensors mainly focus on the structural study of suspended graphene and supporting graphene and are manufactured using traditional technology. Although substantial effort has been exerted, small linear pressure and small pressure tolerance ranges still exist in the sensors. In particular, the sensitivities of the sensors are always in the low level of the same order of magnitude. The development of these sensors has been hampered by factors such as inadequate sensitivity; therefore, new approaches must be explored to further improve their performance.

The post-treatment process can promote rearrangement of the material lattice and clean up the contaminants on the material surface, which is helpful to further improve the sensing performance of devices. The effects of different post-treatment processes on the properties of graphene have been reported. Plasma treatments on graphene surfaces can reduce polymer residue contamination but result in graphene with a defective graphitic structure [23]. New defects, such as amorphous carbon and sp3 bonds, which are generated by the binding of plasma components to graphene during the plasma step, are introduced. These defects change the periodic structure of graphene and reduce its original electrical properties. The removal of polymer residues on the graphene surface has been investigated under various annealing conditions, such as vacuum, nitrogen, hydrogen, and argon atmospheres [24,25]. The results do yield the reduction of polymer residue contamination on graphene by annealing in the aforementioned conditions, but these conditions are strict, and operation is relatively complex for pure gas annealing. Therefore, an easier and more effective method must be developed to improve device performance. The operation of atmospheric annealing is simple and is beneficial in terms of environmental protection and resource saving. Atmospheric annealing has the dual abilities of removing polymer residues and hydrocarbon contaminants from a graphene surface, which gives it the potential to achieve a cleaner graphene surface [26,27]. It is mostly equivalent to oxygen/nitrogen mixture annealing, where the oxygen is likely to bridge the substrate with graphene to promote a better response of graphene to substrate deformation [28]. The same results as pure gas annealing can be expected from atmospheric annealing.

A graphene MEMS pressure sensor with a Wheatston bridge circuit formed from several graphene resistances integrated on a chip, with pressure sensing achieved only by the in-plane compressible properties of a graphene monolayer with exceptional properties [29,30,31], is proposed in this study. The supporting film is thickened in order to enhance the linear response range and pressure-bearing capacity, and the graphene monolayer on the sample is bent repeatedly and annealed in atmosphere at 300 °C to improve the sensing performance. The results show that the annealing in atmosphere enhances the pressure-sensing sensitivity and improves the response speed and linear response range of the sensor, benefiting from the desorption of hydroxyl groups on the graphene surface during annealing. This condition demonstrates that atmospheric annealing at 300 °C enables the graphene monolayer to have the same response effect as pure gas annealing. The performance of the proposed sensor is comparable to and even better than those of previous sensors based on graphene in-plane properties. Our results show that the optimization process of the atmospheric annealing at low temperature can provide a new method for improvement of the sensitivity, response speed, and other characteristics of MEMS sensors and leads it to have potential application prospects in the fields of medicine (e.g., blood pressure monitoring), electronics (e.g., the altimeter of unmanned aerial vehicles), automotive industry (e.g., tire pressure monitoring), and chemical industry (e.g., oil pressure detection).

## 2. Materials and Methods

### 2.1. Device Fabrication

A commercial single-crystal silicon wafer was purchased. The silicon wafer was grown following the directions in [110], and its thickness was 0.5 mm. A graphene monolayer coated by polymethyl methacrylate (PMMA) was a trivial transfer graphene from ACS MATERIAL (XFNANO Co., Ltd., Nanjing, China).

**Figure 1:**
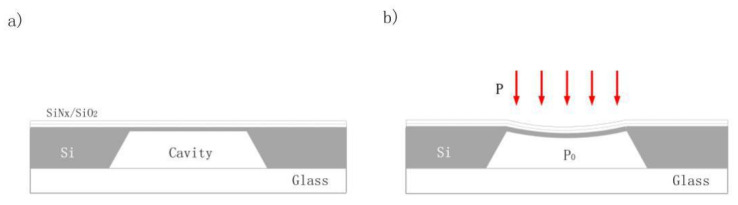
Schematic of the device: (**a**) basic structure of the device; (**b**) status of the device under the application of differential pressure.

A schematic of the device is shown in Figure 1. A multilayer component membrane, which includes silicon, silicon dioxide, and silicon nitride (SiN*x*), was used as the structural element in this pressure-sensing device. As illustrated in Figure 1a, the suspended membrane was poised because the pressure inside and outside the sealed cavity is the same when not disturbed by outside factors. A schematic of the device under testing is shown in Figure 1b. *P*_0_ is the pressure inside the cavity, and *P* is the pressure outside the cavity. When differential pressure is applied, the membrane undergoes deflection and deforms into a concave shape.

**Figure 2:**
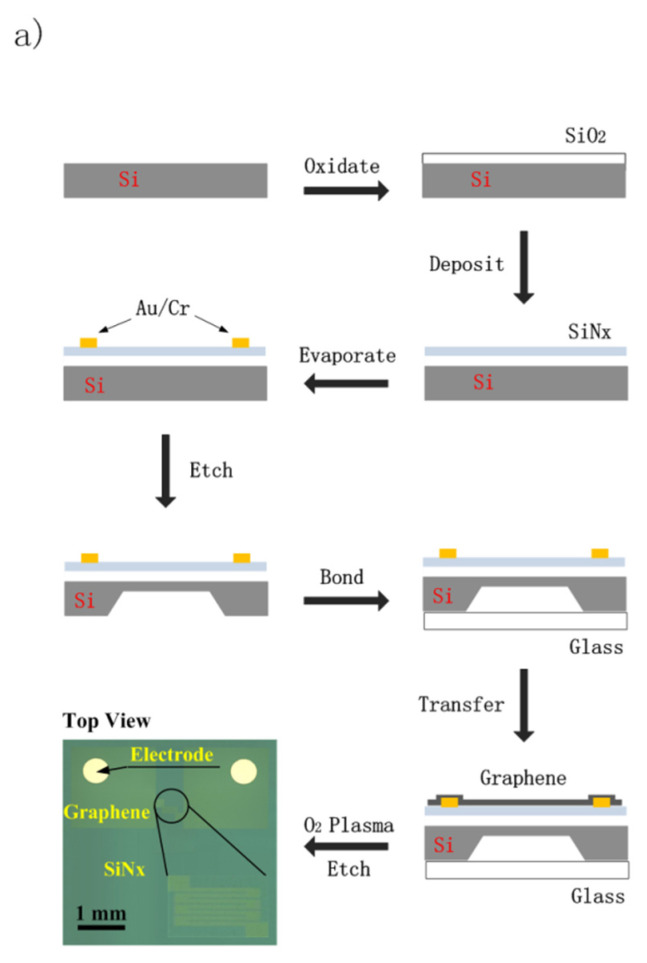

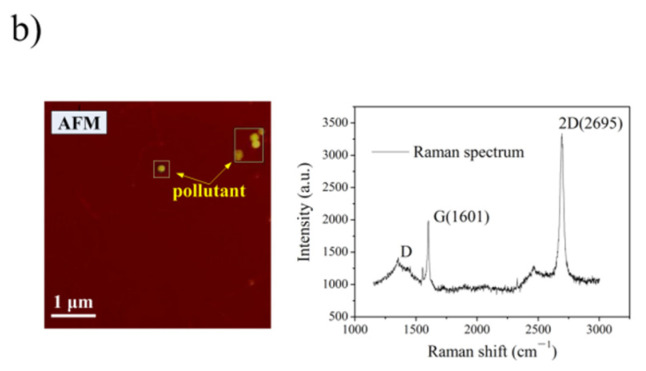
Preparation and characterization of the device: (**a**) technological process for fabricating devices; (**b**) atomic force micrograph and Raman spectrum of graphene.

The technological process for fabricating the device is shown in Figure 2a. The process includes oxidizing, depositing, evaporating, etching, bonding, and imaging graphene. The lateral view in Figure 2a depicts the device structure for every step of the process, and the top view in Figure 2a presents the pattern of graphene on the SiN*x* membrane after etching by O_2_ plasma. From the top view, only the graphene monolayer resistance has the meander pattern. The graphene resistance is above the cavity and acts as a sensitive element.

The devices are fabricated using standard techniques. The silicon nitride membrane was deposited on the oxide with a thickness of 200 nm for electrical isolation after the silicon wafer was thermally oxidized. Its thickness was about 100 nm. Cr (50 nm) and Au (250 nm) were evaporated onto the silicon nitride layer for the contact. The electrode patterning was acquired by photolithography and chemical etching. The devices must possess electromechanical sensing ability. To this end, 1400 μm-wide square cavities defined with photoresistance were etched 450.3 μm deep into the silicon layer at the backside of the wafer. A piece of glass was bonded with the wafer’s backside in order to seal gas in the cavities, which can form a pressure difference inside and outside of them. The graphene monolayer was transferred to the abovementioned substrate. The graphene with PMMA was rinsed in deionized water and picked up with the chip. The chip was baked for 20 min at 100 °C to allow the graphene to adhere to the silicon nitride layer and the gold electrodes. The PMMA was then dissolved by acetone and rinsed with ethyl alcohol and deionized water. The chip was then baked again for 10 min at 50 °C to dry the graphene. To image the graphene, its patterning was defined by photolithography and etched using an O_2_ plasma process.

### 2.2. Material Characterization

In recent years, the atomic force microscope, Raman spectrometer, infrared spectrometer, and X-ray photoelectron spectrometer have become powerful characterization and metrology tools for solid materials at the nanoscale. They are employed to detect the roughness, number of plies, and functional groups of the graphene and to verify its quality on the chip surface.

The atomic force microscope used was a Dimension Edge (Brock Company, Saarbrucken, Germany). This microscope has an atomic resolution with a lateral resolution of 0.1 nm and a longitudinal resolution of 0.01 nm. The Raman spectrometer was an inVia (Renishaw Company, Gloucestershire, UK). The spectrometer was used to test graphene with a laser wavelength of 532 nm and a laser power of 2.4 mW. The infrared spectrometer was a Frontier (PE Company, Waltham, MA, USA). Its spatial resolution was 1.56 μm. A resolution of 4 cm^−1^ was used for spectral testing. The photoelectron spectrometer used was an ESCALAB 250Xi (Thermo Company, Waltham, MA, USA). This instrument has an energy resolution of 0.45 eV/(Ag 3d5/2) and a spatial resolution of less than 3 μm.

The atomic force micrograph and the Raman spectrum of the graphene are shown in Figure 2b. Although the micrograph indicates that the graphene is smooth, a few pollutants are observed on the graphene surface. From the Raman spectrum, the graphene monolayer is verified, and few defects are observed in the graphene.

### 2.3. Measurement

**Figure 3:**
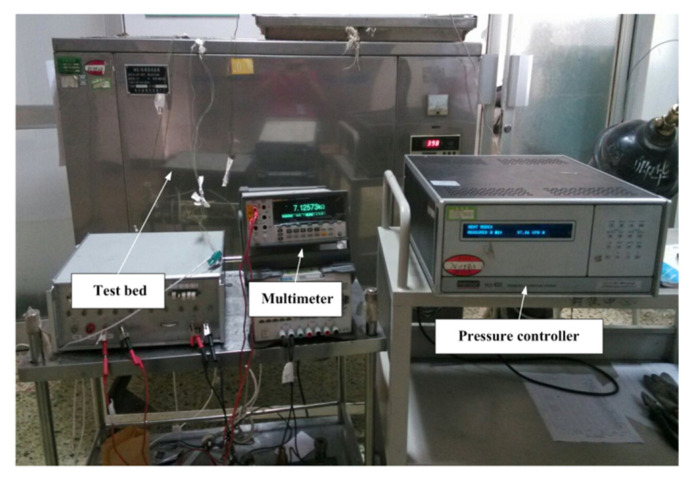
Image of the experimental set-up.

As shown in Figure 3, a MENSOR pressure controller was employed to output the gas pressure that can be directly indicated by the controller. The pressure controller used was a PCS 40G (Mensor Company, San Marcos, TX, USA). The pressure test range was 0–7 MPa. The resolution was up to 5 digits. The accuracy was 0.05%. The response time was about 100 ms. Argon was used, and positive gas pressure can be applied to a device when it operates, causing a pressure difference between the inside and outside of its cavity. The resultant bending of the pressed cavity membrane leads to deformation of the graphene, thereby enabling the sensing ability of the device. The multimeter used was an 8845A (Fluke Company, Everett, WA, USA). This instrument has a 6.5-bit digital resolution and an accuracy as high as 0.0024%; its resistance range is from 10 Ω to 1 GΩ. The multimeter was utilized in combination with the pressure controller to obtain the electromechanical measurement data, and electromechanical measurements were performed for the sample at room temperature following different post-treatment processes, including 300 °C annealing in atmosphere and no annealing.

### 2.4. Simulation

A COMSOL finite element method was used to analyze the strain distribution caused by the differential pressure across the cavity membrane. The simulation results were obtained through a process that included setting geometric dimensions, setting materials, selecting solid mechanical-physical fields, fine meshing, researching, and computing. The whole strain distributions on the cavity membrane were simulated when pressure differences of 0.1 and 0.5 MPa were applied to this membrane. The strain data were correspondingly extracted and analyzed when pressure differences from 0.1 MPa to 0.5 MPa were applied to the cavity membrane.

Density functional theory (DFT) was used to calculate the difference of the charge distribution and Fermi velocity of graphene absorbing pollutants in order to illustrate the effects of annealing on the device properties. A primitive graphene cell was constructed using two carbon atoms. In our study, we considered a 6 × 6 × 1 graphene supercell consisting of 36 1 × 1 × 1 unit cells, and one pollutant, such as a hydroxide radical, was placed on the top of the carbon atom. Approximately 25 Å of vacuum was added to the graphene surface in order to separate the repeated cells along the *c* axis. Calculations were performed using a 2 × 2 *k*-point mesh for the supercell surface and using one mesh point along the *c* direction.

For the difference of the charge distribution, the constructed hydroxy-adsorbed graphene system was optimized to the minimum energy needed to obtain a stable system. For the charge distributions of this system, the graphene retained by removing the hydroxyl group from the system and the hydroxyl group retained by removing the graphene from the system were calculated. The charge distribution of the system was subtracted from the charge distributions of the independent graphene and hydroxyl groups. In this way, the charge changes of the adsorbed hydroxyl graphene relative to the independent, clean graphene are obtained. The Fermi velocity was obtained by calculating the energy band along the path of *K* points in the inverted lattice space of the graphene system using the DFT method.

## 3. Results

As shown in Figure 1, the device has an outer dimension of 4.27 mm × 4.27 mm and a thickness of about 0.9 mm. The inner square cavity has a side length of 1400 μm and a depth of about 450.3 μm. The edge length of the film above the cavity is 1400 μm, and its thickness is about 50 μm. In accordance with the structure of the device, the film above the square cavity plays a major role in sensing.

**Figure 4:**
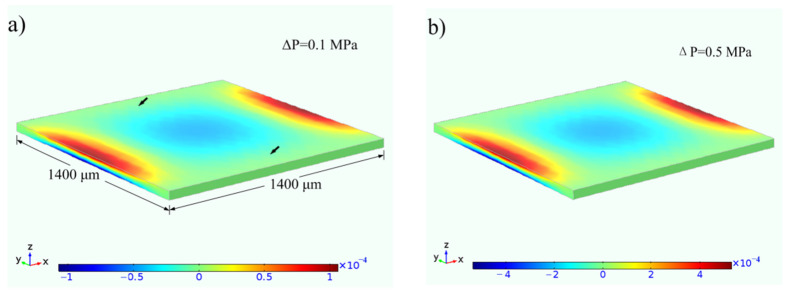
Simulative strain fields for cavity membrane: (**a**) strain $\varepsilon_{xx}$ distribution under the differential pressure with 0.1 MPa; (**b**) $\varepsilon_{xx}$ distribution under differential pressure with 0.5 MPa.

**Figure 5:**
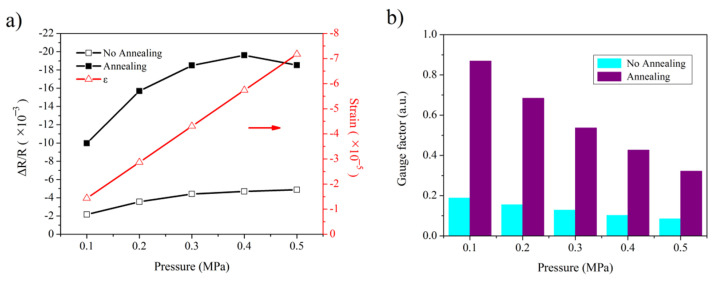
In-plane compressible characteristics of graphene: (**a**) relative changes in single graphene resistance; (**b**) comparison of the gauge factor of the graphene compressed along its plane.

COMSOL simulation was performed using the details provided in Section 2.4 in order to determine the deformation of the suspended membrane above the sealed cavity. The whole strain distributions in the x-axis direction are shown in Figure 4 when pressure differences of 0.1 and 0.5 MPa were applied to this membrane. From Figure 4, tensile and compressive distortions can be observed on the edge of the membrane. The only graphene resistance in Figure 2a is located at the compressive region (marked by either black arrow) in order to explore the sensing characteristic of the graphene contracted by the compressible deformation of the substrate. The strain data at the compressive region were extracted and analyzed when pressure differences from 0.1 MPa to 0.5 MPa were applied to the cavity membrane. The average strain at each pressure level was obtained by averaging a large number of strain values over the region of the single graphene resistance as the sensitive element. Therefore, the averaged strains in the region (marked by a black arrow in Figure 4a) are shown in Figure 5a. The averaged strain increased linearly with the gradual increase in pressure. Subsequent measurements at room temperature were performed for the device with and without annealing in atmosphere at 300 °C in order to compare the performance of the device before and after annealing [32]. The resistance values of a single sensitive graphene resistance before and after pressure application were obtained by applying a pressure controller and monitoring device resistance change with a multimeter. The relative change in the graphene resistance was obtained by dividing the difference in resistances before and after pressure application by the initial resistance. Figure 5a shows the relative change in the graphene resistance at each pressure value. As shown in Figure 5a, the relative changes with only graphene resistance as the sensitive element indicate that its resistance decreases when the graphene is compressed along the plane, which is the exact opposite of graphene in its stretched state. The relative changes exhibit nonlinear growth with the gradual increase in pressure, and that of devices undergoing 300 °C annealing is greater than that of one without annealing at the same pressure. The gauge factor of the in-plane compressible graphene was analyzed using the expression *G* = Δ*R*/*Rε*. The gauge factor for graphene was obtained by dividing the relative change in graphene resistance at each pressure by the corresponding mean strain in Figure 5a. Figure 5b presents the gauge factors of the compressible graphene that are normalized to obtain the degree of difference between the gauge factors of graphene before and after annealing at the same pressure. The gauge factor of graphene decreases nonlinearly, and the degree of difference between the gauge factors of graphene before and after annealing decreases gradually with the increase in pressure. The gauge factor of a device undergoing 300 °C annealing is more excellent than that of one without annealing, and the former is about four times higher than that of the latter.

**Figure 6:**
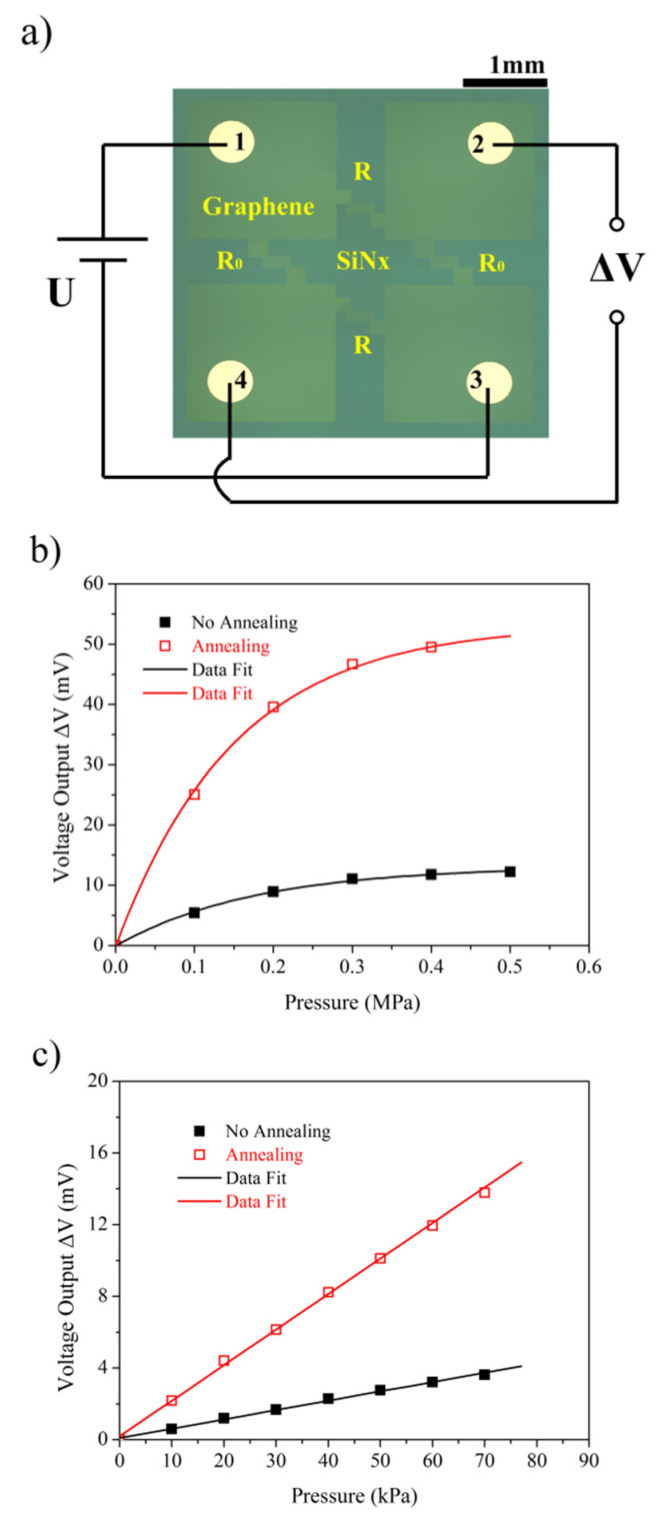
Voltage output as a function of the differential pressure: (**a**) optical image of the proposed sensor; (**b**) in the range of 0 MPa to 0.5 MPa; (**c**) in the range of 0 kPa to 70 kPa.

A new pressure sensor based on the above sensitive element is proposed and presented in Figure 6a to further understand the sensing performance of the device based on the in-plane compressible graphene. The sensor consists of four graphene resistances with meander patterns that are symmetric to the sensor center. Their original resistances are equal, because every meander pattern is the same as that of only a graphene monolayer resistance in Figure 2a. A pair of graphene resistances *R* above the cavity acts as sensitive elements, but the remaining *R*_0_ is not above the cavity and remains constant. As shown in Figure 6a, a DC source and a multimeter can be connected to the circuit so that a Wheatston bridge is formed based on a graphene monolayer with meander patterns A DC source that can provide electricity is connected to input electrodes 1 and 3, and a multimeter that can receive the output signal Δ*V* is connected to output electrodes 2 and 4. The voltage output Δ*V* of the Wheatston bridge can be expressed as Equation (1), where *U* represents the voltage of the DC source.

(1) $$\Delta V=2UR_{0}/(R_{0}+R)-U$$

The electromechanical performance of the proposed sensor can be obtained by the combination of electromechanical measurements of only the graphene monolayer resistance as a sensitive element in Figure 2a, and theoretical analysis for this sensor using Equation (1) [33]. As shown in Figure 6, the derived data at 5 V from the experiment demonstrate that the voltage output increases nonlinearly with the increase of differential pressure in the range of 0 to 0.5 MPa, and the voltage output increases linearly with the increase of differential pressure in the range of 0 to 70 kPa. From Figure 6c, the voltage output of the sensor undergoing 300 °C annealing can reach up to 14.1 mV when the pressure rises to 70 kPa. Its sensitivity, which is about 4.03 × 10^−5^/kPa, is greater than that of the one without annealing. From the performance comparison among the MEMS pressure sensors in Table 1, its performance is comparable to and even better than that of previous sensors. At the same time, the fitting for every data group is provided in Figure 6. The fitting curves are excellent, and the relevant data are listed in Table 2.

**Figure 7:**
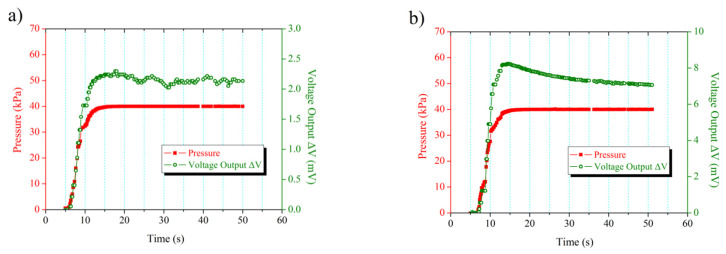
Dynamic response of voltage output with differential pressure: (**a**) without annealing; (**b**) with 300 °C annealing in atmosphere.

The pressed courses are shown in Figure 7 to determine the dynamic response of the sensor. The voltage output increases quickly when the pressure rises to 40 kPa. The voltage output is in exact accordance with the pressure change. Hysteresis phenomena are not observed between the voltage output and the pressure. The voltage output reaches its peak value when the pressure rises to 40 kPa. The voltage output gradually tends to be stable after falling slightly when the subsequent pressure remains constant. The cavity membrane continues to run and subsequently returns to the equilibrium position due to the inertia caused when the pressure reaches its peak. Thus, the phenomenon of a slight decline of the voltage output occurs in accordance with expectations. The fluctuation during tending tends to be stable for the voltage output. As indicated in Figure 7, the fluctuation in voltage output of the sensor that underwent 300 °C annealing is smaller than that of the one without annealing.

**Figure 8:**
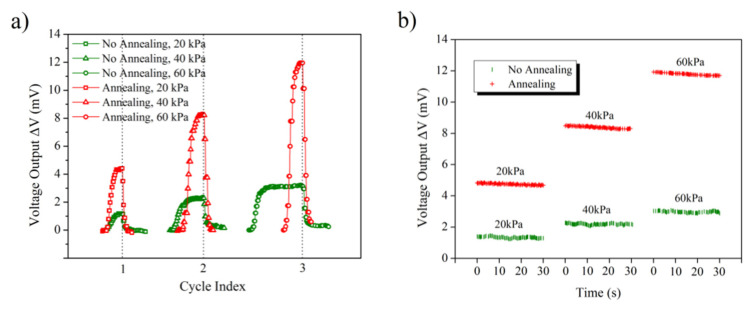
Dynamic voltage output: (**a**) voltage output during cycling tests when switching the differential pressure ON/OFF; (**b**) voltage output versus time under differential pressure with step increase from 20 kPa to 60 kPa (20 kPa per step).

Reversible performance of the sensor can be obtained for the device in Figure 2a by switching the pressure in three cycles of ON/OFF. The discrepancy is illustrated in Figure 8a. Each switching cycle is performed by turning the controller on/off with fixed differential pressure. When the controller is turned off, the strain on the cavity membrane is relaxed because of the quick venting of the gas line. As shown in Figure 8a, the response speed of annealed sensor to gas pressure is faster than that of the nonannealed sensor. Measurements of pressure maintenance are made at varying differential pressures across the membrane, as shown in Figure 8b. The voltage output increases in distinct steps when the strains applied on the graphene resistances as sensitive elements are increased. These defined steps show the good response of the piezoresistive effect of graphene to the membrane deformation. From Figure 8b it can be seen that the effect of pressure maintenance is good for every step, although slight fluctuations of the voltage outputs are observed in most steps.

## 4. Discussion

**Figure 9:**
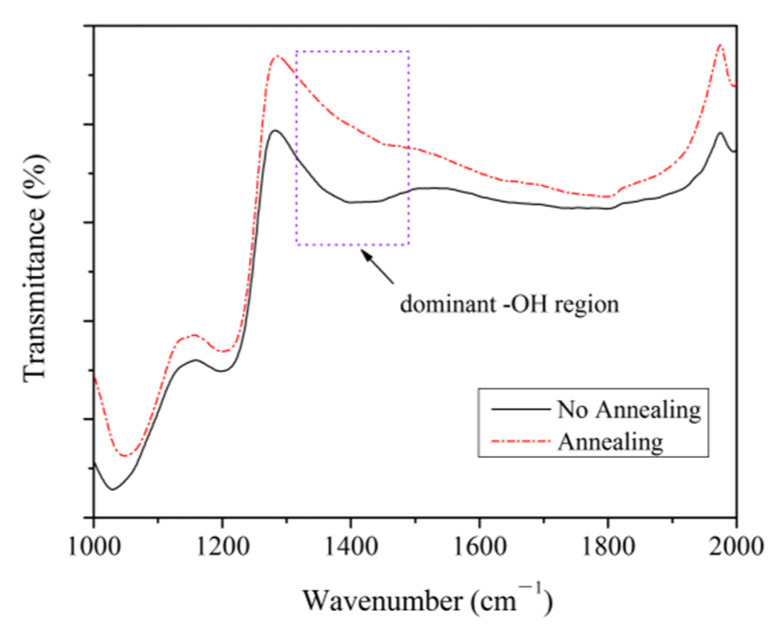
Comparison of the infrared spectra of the graphene monolayer on the substrate with and without annealing.

From the data obtained above, it can be determined that the performance of the sensor undergoing 300 °C annealing is more excellent than that of the one without annealing. The samples are contaminated frequently in the process device fabrication. The pollutants, such as hydrogen ions and hydroxide radicals, may be absorbed on the graphene surface when all types of chemical reagents are applied. Figure 9 shows the comparison of the infrared spectra of the graphene monolayer on the samples with and without annealing. A difference of around 1400 cm^−1^ is observed in the infrared spectra. The spectrum line without annealing has a more prominent bulge than that with annealing. The broad bulge of the spectrum line without annealing is most likely the result of the interaction between the major and minor groups around 1400 cm^−1^. The sample surface contains water molecules, because the sample was rinsed with deionized water in the process or remained in the air. The hydroxyl radicals absorbed on the graphene surface may be formed by the interaction between water molecules attached to the graphene surface and oxygen molecules in air before annealing [34]. The water molecules attached to the graphene surface are removed during low-temperature annealing [35], which results in the loss of hydroxyl functional groups. Theories and experiments prove that the infrared absorption peak of the bending vibration of the hydroxyl group is near 1400 cm^−1^ [36], so the broad bulge in the blue box in Figure 9 may be dominated by the hydroxyl group. The bulge of the spectrum line with annealing in the blue box disappears, which is probably caused by the desorption of hydroxyl groups on the graphene surface after low-temperature annealing.

**Figure 10:**
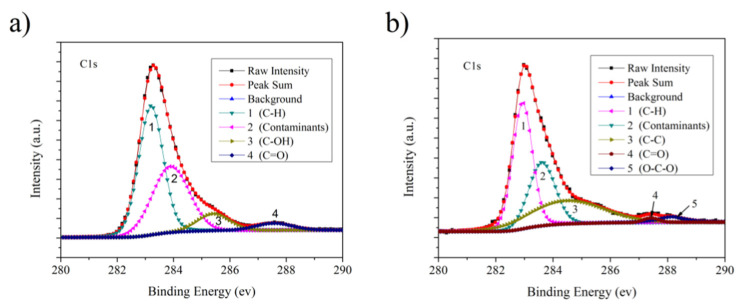
Typical high-resolution XPS spectra of the C1s region of graphene on the sample: (**a**) without annealing; (**b**) with annealing.

Figure 10 shows the high-resolution XPS spectra of the C1s region of graphene on the sample in order to further determine the situation of the functional groups on the graphene surface. The deconvoluted, high-resolution XPS C1s region spectra of graphene on the sample without annealing are shown in Figure 10a. As shown in Figure 10a, the deconvoluted C1s peaks at 1, 2, 3, and 4 show peak binding energies of 283.2, 283.9, 285.4, and 287.6 eV, which correspond to C–H [37], contaminants such as hydrocarbon or carbon element, C–OH [38], and C=O [39], respectively. However, the deconvoluted C1s peaks at 1, 2, 3, 4, and 5 with binding energies of 282.9, 283.6, 284.5, 287.4, and 288.1 eV after annealing are assigned to the C–H, contaminants, C–C [40], C=O, and O–C–O [41], of the graphene on the sample, respectively (Figure 10b). Some hydroxyl groups are adsorbed onto the graphene before annealing, but these hydroxyl groups disappear, and a large number of unsaturated C–C bonds appear in graphene after annealing. This finding is consistent with the speculated results of infrared spectroscopy, which shows that the hydroxyl on the graphene surface can be desorbed, and the electrical properties of graphene can be improved by annealing. Each peak position of C1s is slightly shifted after annealing [42]. The rearrangement of lattice defects or functional groups results in the changes in peak positions during annealing.

**Figure 11:**
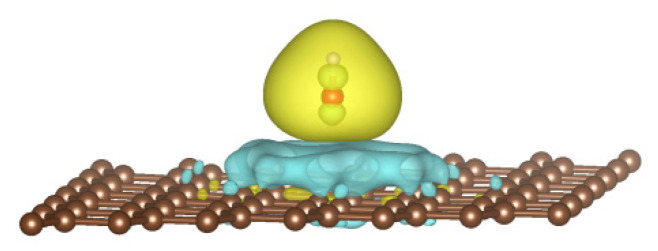
Difference in the charge distribution of graphene absorbing the hydroxide radical marked by red and white.

Figure 11 shows the difference in to charge distribution of the graphene adsorbing hydroxyl group by the DFT method in Section 2.4. The blue regions represent the charge loss, and the yellow regions represent the charge augmentation. The partial electron charges of graphene transfer to the hydroxyl group. The electrical performance of graphene is reduced due to the hydroxyl groups on graphene restraining the partial electron charges of graphene. The hydroxyl group adsorbed on the graphene is equivalent to a defect embedded in the graphene. The defect has a potential well. The potential well tends to trap graphene electrons clustered near the hydroxyl group. This condition prevents them from escaping the hydroxyl group and reduces the number of freely moving electrons in the graphene. This defect has a scattering effect on the graphene carriers passing through itself, which leads to a decrease in the mobility of graphene carriers. The hydroxyl group adsorbed to the graphene surface breaks the periodic structure of the single-crystal graphene, and the large amount of hydroxyl groups adsorbed to the graphene changes the previously clean single-crystal graphene system into a similar polycrystalline system. Grain boundaries in the polycrystalline system inevitably block the movement of graphene carriers, resulting in a remarkable decrease in the mobility of carriers. These conditions are all possible factors that reduce the electrical performance of graphene materials. The desorption of hydroxyl groups leads to the periodic improvement of the graphene lattice after low-temperature annealing for the graphene-adsorbing hydroxyl groups. The lack of defects and grain boundaries restores the high mobility of carriers in graphene, which improves the electrical performance of the graphene materials. The above analysis shows that the high performance of the annealed sample mostly benefits from the desorption of hydroxyl groups on the graphene surface during annealing.

From the above-mentioned experimental results, the sensitivity of annealed graphene is higher than that of unannealed graphene, and the difference between the two sensitivities tends to decrease with the increase in deformation. In accordance with Boltzmann’s theory, this phenomenon can be explained by the relative change in the Fermi velocities of the same material under different deformation conditions. The unannealed graphene, which has a hydroxyl group adsorbed to its surface, has a low initial Fermi velocity. The graphene surface becomes cleaner with hydroxyl desorption, and the cleaner graphene has a higher initial Fermi velocity after annealing. The difference between the initial Fermi velocities of the two graphene systems results in the different levels of sensitivity.

A theoretical model using a linearized Boltzmann transport equation can accurately describe the charge carrier density and mobility in the strained graphene and the gauge factor [43]. The charge carrier density in graphene is approximately proportional to the inverse of the squared Fermi velocity:

(2) $$N_{e}(\varepsilon )\sim 1/v_{F}{\left(\varepsilon \right)}^{2}$$

Considering the scattering mechanism of the defects, the dependence of the mobility on the Fermi velocity is given by Equation (3).

(3) $$\mu_{e}(\varepsilon )\sim v_{F}{\left(\varepsilon \right)}^{4}$$

Thus, the resistivity is approximately proportional to the inverse of the squared Fermi velocity [44]:

(4) $$\rho_{e}(\varepsilon )\sim 1/v_{F}{\left(\varepsilon \right)}^{2}$$

The resistance of a strained graphene can be defined as Equation (5).

(5) $$R=\rho \frac{L^{\prime }}{W^{\prime }}=\frac{1}{2qN_{e}\mu_{e}}\frac{(1+\varepsilon_{xx})L}{(1+\varepsilon_{yy})W}$$

where $\varepsilon_{xx}$ and $\varepsilon_{yy}$ are the strain components in the x and y directions of the graphene, respectively. *q* is the basic unit of charge. $L^{\prime }$ and $W^{\prime }$ are the length and width change in the strained graphene.

The resistance is further expressed as

(6) $$R\sim \frac{1}{v_{F}{\left(\varepsilon \right)}^{2}}\frac{(1+\varepsilon_{xx})L}{(1+\varepsilon_{yy})W}$$

Therefore, the relative change in strained graphene resistance is expressed as

(7) $$\frac{\Delta R}{R_{0}}=\frac{R-R_{0}}{R_{0}}=\frac{v_{F}{\left(0\right)}^{2}}{v_{F}{\left(\varepsilon \right)}^{2}}\frac{\left(1+\varepsilon_{xx}\right)}{\left(1+\varepsilon_{yy}\right)}-1$$

The gauge factor can reflect the sensitivity of the graphene material. The gauge factor can be determined using Equation (8).

(8) $$G=\left|\frac{\Delta R}{R_{0}\varepsilon }\right|$$

**Figure 12:**
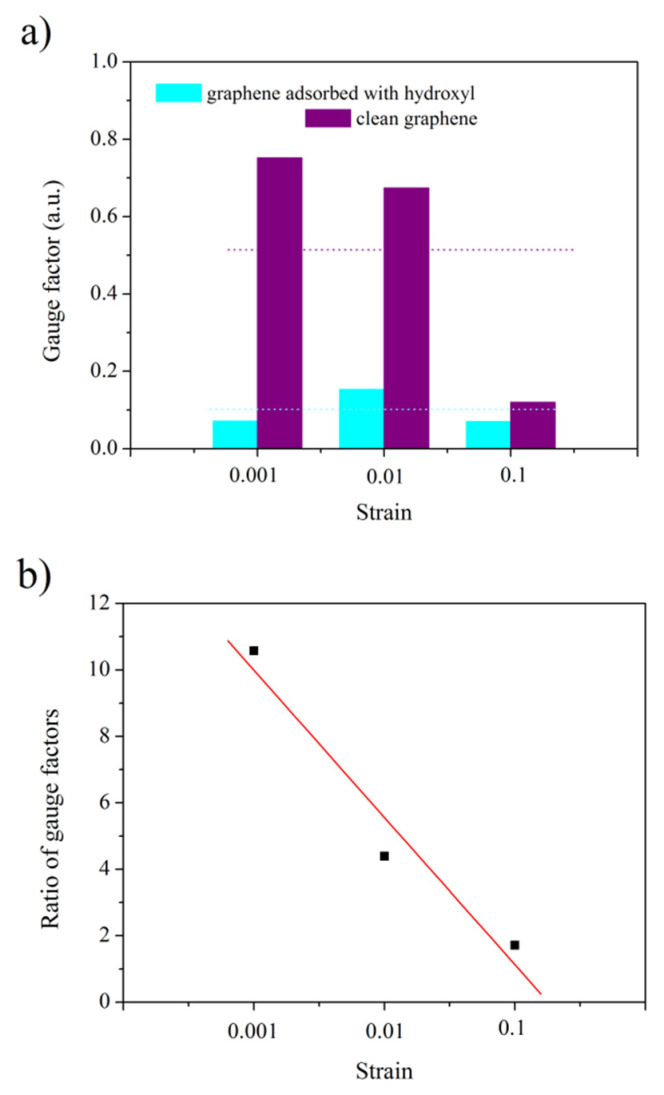

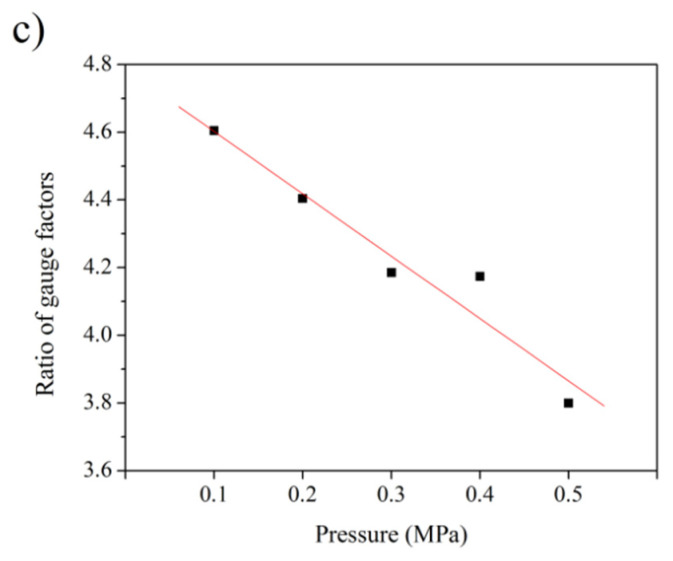
Comparison of the gauge factors between the graphene adsorbed with hydroxyl and clean graphene: (**a**) gauge factors; (**b**) ratio of gauge factors from (**a**); (**c**) ratio of gauge factors from Figure 5b.

The gauge factor of the graphene system under specific deformation can be obtained by calculating the Fermi velocities for the original and deformed graphene systems. The Fermi velocity to be introduced into the formula is obtained by calculating the energy band along the path of *K* points in the inverted lattice space of the graphene system using the DFT method. Therefore, every gauge factor in Figure 12a can be obtained from the theoretical model.

Figure 12a shows the normalized gauge factors for obtaining the degree of difference between the gauge factors of clean graphene and graphene adsorbing a hydroxyl group. As shown in Figure 12a, the sensitivity of the clean graphene is higher than that of graphene adsorbing a hydroxyl group under the same strain. The purple dotted line represents the average gauge factor of clean graphene, and the blue dotted line represents the average gauge factor of graphene adsorbing a hydroxyl group. The average gauge factor of clean graphene is about 5 times higher than that of graphene adsorbing a hydroxyl group. Figure 12b shows the ratio of the theoretical gauge factors of two graphene materials in Figure 12a under the same strain. With the increase in strain, the ratio of the two gauge factors gradually decreases. The theoretical results indicate that the difference between the sensitivities of the clean graphene and the graphene adsorbing a hydroxyl group gradually decreases with the increase in strain. Figure 12c shows the ratio of the gauge factors of annealed and unannealed graphene under the same pressure from Figure 5b. Considering that the state of the device material barely changes before and after low-temperature annealing, the deformation of the device is the same when the device is subjected to the same pressure before and after annealing [45]. This condition results in the same strain to the graphene on the device surface before and after annealing at the same pressure. In accordance with the gauge factor formula, the ratio of the gauge factors of graphene with the same strain will cancel out the same strain. Therefore, Figure 12c shows only the ratio of the relative change in graphene resistance measured before and after annealing under the same pressure conditions, which indicates that the ratio of gauge factors in Figure 12c has no relationship with the borrowed simulation strain and is entirely the result of experimental data calculation. As shown in Figure 12c, the ratio of the two gauge factors gradually decreases with the increase in pressure. This condition indicates that the experimental differences between the sensitivities of graphene before and after annealing gradually decreases with the increase in pressure. The above results show that the prediction made by the theoretical model is in agreement with the experimental results. Thus, the sensitivity of graphene annealed in atmosphere at 300 °C is higher than that of unannealed graphene under the conditions of small deformation, while it no longer has a remarkable advantage over that of unannealed graphene under the conditions of large deformation.

## 5. Conclusions

In this study, we used MEMS standard technology to propose a pressure sensor based on the in-plane compressible characteristic of monolayer graphene and thoroughly investigate its performance before and after atmospheric annealing at 300 °C. The performance metrics, including sensitivity, response speed, and linear response range, were markedly improved by atmospheric annealing. Atmospheric annealing at 300 °C enhanced the pressure-sensing sensitivity by 4 times compared with the pristine graphene without annealing, which can be proven by the differential degree of the gauge factors of graphene before and after annealing. The response speed of the annealed sensor to gas pressure was faster than that of the nonannealed sensor. The linear response range of 0–70 kPa is an improvement over those of previous sensors. Simultaneously, the research shows the effective response of the in-plane piezoresistive effect of monolayer graphene to gas pressure, and that the performance of the sensor undergoing 300 °C annealing is more excellent than the one without annealing, which benefits predominantly from the desorption of hydroxyl groups on the graphene surface during annealing. The sensitivity of graphene annealed in atmosphere at 300 °C is higher than that of the unannealed graphene under the conditions of small deformation, but it no longer has a remarkable advantage over that of unannealed graphene under the conditions of large deformation. Our results provide new insights into obtaining high performance of MEMS devices based on 2D sensitive materials.

## Figures and Tables

**Table 1:** Performance comparison among the MEMS pressure sensors.

Group	Device Material	Sensitivity (kPa^−1^)	Pressure Range (kPa)	Linear Range (kPa)	Year
Smith [18]	Monolayer graphene	2.96 × 10^−5^	40–100	60–90	2013
Zhu [19]	Multilayer graphene	3.33 × 10^−5^	0–70	0–50	2013
Wang [20]	few-layered graphene	1.94 × 10^−5^	0–60	0–40	2016
Liu [21]	Monolayer graphene	1.86 × 10^−5^	0–70	---	2020
Lin [22]	Monolayer graphene	2.87 × 10^−5^	0–80	---	2019
This paper	Monolayer graphene	4.03 × 10^−5^	0–500	0–70	2022

**Table 2:** Relevant data of the fitting curves in Figure 6.

Category	Expression	*R* ^2^	*R*	Pressure Range
No annealing	*y* = −13.24632exp(−*x*/0.17623) + 13.16728	0.99826	---	0 MPa to 0.5 MPa
Annealing	*y* = −53.51491exp(−*x*/0.15024) + 53.31026	0.99928	---	0 MPa to 0.5 MPa
No annealing	*y* = 0.05213*x* + 0.09522	---	0.99817	0 kPa to 70 kPa
Annealing	*y* = 0.19839*x* + 0.19942	---	0.99960	0 kPa to 70 kPa

## Data Availability

Data is contained within the article.
